# Exploring biomedical ontology mappings with graph theory methods

**DOI:** 10.7717/peerj.2990

**Published:** 2017-03-02

**Authors:** Simon Kocbek, Jin-Dong Kim

**Affiliations:** 1 Database Center for Life Science, Research Organization of Information and Systems, Tokyo, Japan; 2 Kinghorn Centre for Clinical Genomics, Garvan Institute of Medical Research, Sydney, NSW, Australia; 3 Department of Computing and Information Systems, University of Melbourne, Melbourne, Victoria, Australia

**Keywords:** Ontology evolution, Biomedical ontology, Ontology mappings, Semantic web, Graph theory

## Abstract

**Background:**

In the era of semantic web, life science ontologies play an important role in tasks such as annotating biological objects, linking relevant data pieces, and verifying data consistency. Understanding ontology structures and overlapping ontologies is essential for tasks such as ontology reuse and development. We present an exploratory study where we examine structure and look for patterns in BioPortal, a comprehensive publicly available repository of live science ontologies.

**Methods:**

We report an analysis of biomedical ontology mapping data over time. We apply graph theory methods such as Modularity Analysis and Betweenness Centrality to analyse data gathered at five different time points. We identify communities, i.e., sets of overlapping ontologies, and define similar and closest communities. We demonstrate evolution of identified communities over time and identify core ontologies of the closest communities. We use BioPortal project and category data to measure community coherence. We also validate identified communities with their mutual mentions in scientific literature.

**Results:**

With comparing mapping data gathered at five different time points, we identified similar and closest communities of overlapping ontologies, and demonstrated evolution of communities over time. Results showed that anatomy and health ontologies tend to form more isolated communities compared to other categories. We also showed that communities contain all or the majority of ontologies being used in narrower projects. In addition, we identified major changes in mapping data after migration to BioPortal Version 4.

## Introduction

Ontologies are used for tasks such as the standardization of terminology, the verification of data consistency, and the integration of heterogeneous databases. Ontologies have been actively applied to areas including, but not limited to Biology and Medicine (Whetzel et al., 2011), Crisis Management (Liu, Shaw & Brewster, 2013), Information Security (Vorobiev & Bekmamedova, 2010), and Software Engineering (Happel & Seedorf, 2006). In this work, we focus on the area of life sciences, where ontologies are commonly used in tasks such as annotation of gene products and proteins in different databases (Magrane & Consortium, 2011; Flicek et al., 2013; The Gene Ontology Consortium, 2015), or structuring and searching data sources (Doms & Schroeder, 2005).

Life science *ontology mappings* identify existing concepts with similar meaning. These ontology mappings are useful in tasks like finding new annotations, supporting other data integration methods, combining related ontologies, or ontology reuse. When ontologists build new ontologies they often search for existing ontologies to avoid redundancy of concepts as recommended, for example, by the OBO Foundry principles (Smith et al., 2007). Identifying ontology mappings and understanding how ontologies relate is a critical step in integrating data and applications that use different ontologies ( Ghazvinian, Noy & Musen, 2009).

In this paper we analyse and evaluate NCBO BioPortal (Whetzel et al., 2011) ontology mappings. BioPortal is a comprehensive publicly available repository of live science ontologies. It offers several functionalities, for example, browsing and searching for ontologies or defining ontology mappings. BioPortal ontologies are frequently being updated with newer versions. As a result, ontologies may contain new concepts, relations or ontology mappings, or contain other modifications. In scientific community, these changes are often referred to as the *evolution of ontologies* (Kirsten et al., 2011; Hartung, Groß & Rahm, 2013). To help ontology engineers in understanding how ontologies overlap and evolve, we use concepts from graph theory to identify clusters of BioPortal ontologies (i.e., *communities*) that tend to overlap more often than others. Please note that in this paper we use the word *community* for a set of ontologies that tend to overlap. In contrast, the OBO (Open Biomedical Ontologies) project defines a community as a set of ontologies that work together and reduce mutual overlap. We also recognize *hub* ontologies, i.e., ontologies that connect many other ontologies/communities. Since BioPortal data often changes (e.g., new ontologies or versions of ontologies are uploaded or new mappings are defined), we analyse the mapping data at different time points. We propose an alignment of similar communities, define stable communities and perform a time transition analysis. Our work aims to answer questions like “In my area of interest, what ontologies already exist and how are they related to each other?” or “In my area of interest, which sub-areas are stable and which are not in terms of ontology development?”. Answering these questions can assist in tasks like ontology reuse and development. The results of the data gathered and analysed at five different time points are presented.

### Related work

Related work can be categorized in the following (often overlapping) two groups: (1) analysis of ontology mappings and (2) evolution of ontologies. Below we introduce the most relevant papers from these groups.

Similar to our work, Ghazvinian et al. (2009) performed analysis of BioPortal mappings. The goal of their work was to learn more about the characteristics of the ontologies and the relationships between them. As a result, they produced graphs of subsets of biomedical ontologies. Although Ghazvinian’s work addresses a similar problem, our work uses a different approach (i.e., modularity analysis as described in the Methods section) to cluster ontologies in communities and identify hub ontologies. In addition, we also analyse transition between different time points, and identify stable and similar communities. As a result, we offer our work as a supplement to Ghazvinian’s findings about biomedical ontologies and their mappings.

Changes in ontologies have been previously studied and tools such as GOMMA (Kirsten et al., 2011) have been developed. The GOMMA framework provides a scalable and comprehensive infrastructure to analyse large life science ontologies and their evolution. Hartung, Groß & Rahm (2013) investigates evaluation of ontology mappings for different versions of the same ontology. However, as far as we know, no previous work analysed evolution of overlapping communities of ontologies as we do in this paper.

This work is partly a result of our BioHackathon activities (Katayama et al., 2014) and prior work (Kocbek, Perret & Kim, 2012), where we produced a graph representation of BioPortal ontologies. In our later work (Kocbek et al., 2013) we performed initial analysis of differences between two graphs. There are several new contributions in this paper compared to the previous work. First, in the previous work, a preliminary investigation with a basic analysis of mapping data at only two time points was performed. Limited data did not allow detailed trend analysis. On the other hand, data gathered at five time points offers a comprehensive analysis of identified communities (e.g., identifying *stable* and *similar* communities), which is the focus of this paper. We also perform analysis of project and category data and discuss alignment with identified communities. In addition, we try to validate generated clusters with information found in MEDLINE abstracts (Miller, Lacroix & Backus, 2000).

## Data and Methods

### Data

To investigate the state and evolutionary change of biomedical ontologies, we need a comprehensive collection of ontologies. We have chosen to investigate BioPortal data since it is widely recognized as a comprehensive repository of biomedical ontologies. Parts of this and the next section summarize our previous paper with added information.

The data (Supplemental Information 1) was gathered at the following five time points: October 2012, February 2013, August 2013, December 2013 and July 2014. Currently BioPortal contains more than 400 ontologies grouped into 41 categories (e.g., Health, Anatomy, Cell). To perform the analysis, the following data had to be collected through BioPortal RESTful web services for all time points: the ontology’s full name (e.g., Gene Ontology), the ontology’s name abbreviation (e.g., GO), and the number of mappings from/to the ontology. Since the BioPortal RESTful interface changed after August 2013, we gathered the following additional data only for the first three versions of our visualizations: ontology statuses (e.g., production) and ontology versions (e.g., alpha). To analyse identified communities, we also collected number of projects and categories that community members belong to.

Our analysis depends on mapping information in BioPortal. The BioPortal web page describes mappings as:

“*Mappings are associations between two or more terms in different ontologies. This association typically, but not always, represents a degree of similarity between the terms. The author of the mapping defines the semantics of a particular mapping. It is also usual for a mapping to be bi-directional, but again, this is not required. The mapping author defines directionality*.”

We collected the number of all mappings between ontology pairs. The following three types of mappings are supported (please note that no information about mapping types was gathered for our current analysis):

• NCBO mappings are periodically calculated with a computer algorithm. The algorithm finds mappings for terms with close lexical match or mappings for terms with the same URI from different ontologies. The majority of the mappings is from this group.
• Unified Medical Language System (UMLS) mappings link terms with the same UMLS concept unique identifier (CUI) or terms from the UMLS MRMAP.RRF data.
• Mappings between ontology terms related by an OBO (Open Biological and Biomedical Ontologies) xref property.

### Detecting communities and hub ontologies

In the next step, pre-processing of the data was performed. For all ontology versions prior December 2013, we removed the following: (1) ontologies with the retired or alpha status, (2) ontologies that contain the keyword *test* in their full name, and (3) restricted or private ontologies. From data gathered in December 2013 and July 2014 we removed summary ontologies (i.e., they contain the *summaryOnly*  = *true* field). The filtered data was then processed with Gephi (Bastian, Heymann & Jacomy, 2009), an open source tool for graph analysis and visualization. Gephi was chosen because it’s free, platform independent, and several graph and node properties can be calculated. The input file format contained the following three fields:

(1) *fromOntology*: the name of the source ontology,

(2) *toOntology*: the name of the target ontology

(3) *numberOfMappings*: number of directed mappings between source and target ontology.

To identify communities of densely overlapped ontologies, we applied Gephi’s *Modularity Analysis* (also called *Community Detection*) to the data. Modularity Analysis (MA) is a measure of structure in graphs. Gephi implements Louvain method (Blondel et al., 2008) for MA, which is the fastest and most accurate method in terms of modularity score (Aynaud & Guillaume, 2010). Graphs with a high MA score have sophisticated internal structure with separate communities of densely connected nodes inside the communities and sparse connection across communities. To separate communities as much as possible, we ran MA with different resolution parameter values (ranging from 0.8 to 1.2) until the highest MA score for each graph was calculated. The resolution parameter controls number of communities but it results in different MA score. The numbers of mappings between ontologies were used as weights in computing MA scores.

Next, we used the Gephi’s *Betweenness Centrality* (BC) metric (Freeman, 1977) to identify “hub” ontologies. BC is a measure of the frequency of occurrence of a particular node in all the shortest paths between any two nodes. A BC value is calculated for ach node where nodes with a higher BC value play an important role in connecting other ontologies and communities of ontologies.

### Validating the communities with MEDLINE

We used information from MEDLINE abstracts (Miller, Lacroix & Backus, 2000), to analyse how often ontologies from same/different communities found in our latest time point appear together in scientific literature. The goal of this exercise was to validate the clusters with external information.

We downloaded the 2016 version of MEDLINE in XML format and developed an algorithm to find pairs of ontology names in all abstracts published before August 2014 (our latest version of the graph is July 2014). Ontology names and abstracts were transformed to lower case characters before the comparison. Simple exact string matching was used to look for ontology names mentions. For example, in the following text “… we introduce GoPubMed, a web server which allows users to explore PubMed search results with the Gene Ontology…”, Gene Ontology would be identified.

### Aligning communities

Running the community detection algorithm at five time points provides us with different number of communities for each time point. Our previous research (Kocbek et al., 2013) showed that most communities at the time point *t* contain at least some ontologies from the previous time point *t* − 1. The challenge is to align similar communities to compare graphs at multiple time points. With aligned communities we can identify communities that changed their size, new communities, or disappearing communities.

There are several ways to find similar communities in evolving graphs (Freeman, 1977; Hopcroft et al., 2004) and no method suits all problems. So, how do we decide when two identified communities are similar? For p‘ractical reasons we wish to make this decision as simple as possible. Probably the simplest definition would be that two communities are more similar when they share the highest number of nodes compared to other pairs of communities. However, this simple method has a drawback. It has been proven that already small graph changes may affect MA score of Louvain algorithm (Aynaud & Guillaume, 2010). Since BioPortal represents a dynamic repository, it is likely that some identified communities represent unstable communities.

Therefore, we wish to use a more stable method for identifying similar communities. We expect that ontologies with the highest BC scores play an important role in BioPortal as they will likely stay in the repository in the future. We call these ontologies *community core* ontologies. In addition, we should consider ontologies that are not shared between two communities. Based on these issues, we first define several terms that are explained in the following paragraphs.

Let us imagine that we identified two groups of communities where group *C1* contains communities identified at time point *t*1 and *C2* contains communities identified at time point *t*2 (*t*2 > *t*1). First, we define the *importance score of ontology o* as:

\begin{eqnarray*}{I}_{o}= \frac{{BC}_{o,t1}+{BC}_{o,t2}}{2} \end{eqnarray*}

where *BC*_*o*,*t*1_ and *BC*_*o*,*t*2_ represent BC scores for ontology *o* at time points *t*1 and *t*2 respectively.

Next, we define a *similarity score SC*_*cx*,*cy*_ between two communities *cx* ∈ *C*2 and *cy* ∈ *C*1. The similarity score is based on a weighted version of the Dice coefficient (Dice, 1945) and represents a value between 0 and 1. We calculate the similarity score as:

\begin{eqnarray*}{SC}_{cx,cy}= \frac{\sum _{o\in O}{I}_{o}}{\sum _{o\in O}{I}_{o}+\sum _{o\in N}{I}_{o}} \end{eqnarray*}

where *O* represents a set of overlapping ontologies, and *N* represents a set of non-overlapping ontologies found in *cx* or *cy*.

We also define the *closest community to cx*  ∈*C*1 (i.e.,  *CC*_*cx*_ as the community *cy* ∈ *C*2 with the highest similarity score when comparing to *cy*:

\begin{eqnarray*}{CC}_{cx}=cy\text{with Max}\{{SC}_{cx,cy}\}. \end{eqnarray*}

**Figure 1:**
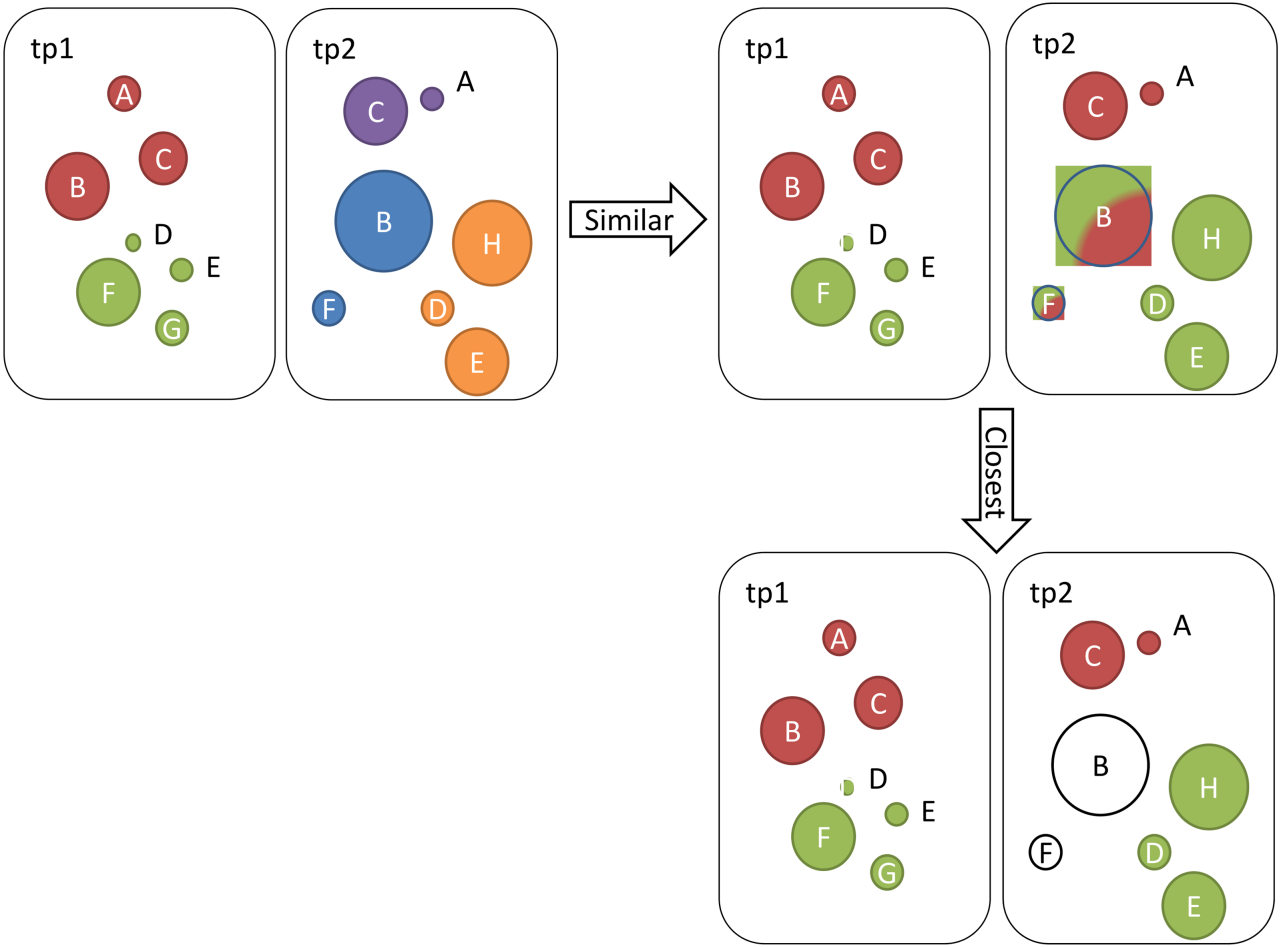
Illustration of identified communities at two different time points tp1 and tp2. Ontologies (circles) that belong to the same community are coloured the same.

Let us illustrate these definitions on an example where we identified five communities at two different time points. At the first time point we identified two communities and at the second time point we identified three communities. Ontologies in each community and their BC scores are presented in Table 1. Figure 1 illustrates the steps described below.

**Table 1:** An example illustrating identified communities at two different time points.

tp1 (2 communities)			tp2 (3 communities)		
Com	Ont	BC	Com	Ont	BC
c1	A	2	c3	B	6
	B	4		F	2
	C	3	c4	A	1
c2	D	0		C	4
	E	1	c5	D	2
	F	4		E	4
	G	2		H	5

To calculate similarity scores between pairs of communities, we first calculate importance of ontologies:

\begin{eqnarray*}& & {I}_{\mathrm{A}}=(2+1)/2=3/2;{I}_{\mathrm{B}}=(6+4)/2=5;{I}_{\mathrm{C}}=(3+4)=7/2;{I}_{\mathrm{D}}=(0+2)/2=1; \end{eqnarray*}

\begin{eqnarray*}& & {I}_{\mathrm{E}}=(4+1)/2=5/2;{I}_{\mathrm{F}}=(4+2)/2=3;{I}_{G}=(0+2)/2=1,{I}_{\mathrm{H}}=(0+5)/2=5/2. \end{eqnarray*}

Next, we calculate the similarity score values for pairs of communities as follows:

\begin{eqnarray*}& & S{C}_{\mathrm{c3,c1}}={I}_{\mathrm{B}}/({I}_{\mathrm{B}}+{I}_{\mathrm{A}}+{I}_{\mathrm{C}}+{I}_{\mathrm{F}})=10/26\approx 0.39 \end{eqnarray*}

\begin{eqnarray*}& & S{C}_{\mathrm{c3,c2}}={I}_{\mathrm{F}}/({I}_{\mathrm{F}}+{I}_{\mathrm{B}}+{I}_{\mathrm{D}}+{I}_{\mathrm{E}}+{I}_{\mathrm{G}})=0.24 \end{eqnarray*}

\begin{eqnarray*}& & S{C}_{\mathrm{c4,c1}}=({I}_{\mathrm{A}}+{I}_{\mathrm{C}})/({I}_{\mathrm{A}}+{I}_{\mathrm{C}}+{I}_{\mathrm{B}})=0.5 \end{eqnarray*}

\begin{eqnarray*}& & S{C}_{\mathrm{c4,c2}}=0/({I}_{\mathrm{A}}+{I}_{\mathrm{C}}+{I}_{\mathrm{D}}+{I}_{\mathrm{E}}+{I}_{\mathrm{F}}+{I}_{\mathrm{G}})=0 \end{eqnarray*}

\begin{eqnarray*}& & S{C}_{\mathrm{c5,c1}}=0/({I}_{\mathrm{A}}+{I}_{\mathrm{B}}+{I}_{\mathrm{C}}+{I}_{\mathrm{D}}+{I}_{\mathrm{E}}+{I}_{\mathrm{H}})=0 \end{eqnarray*}

\begin{eqnarray*}& & S{C}_{\mathrm{c5,c2}}=({I}_{\mathrm{D}}+{I}_{\mathrm{E}})/({I}_{\mathrm{D}}+{I}_{\mathrm{E}}+{I}_{\mathrm{F}}+{I}_{\mathrm{G}}+{I}_{\mathrm{H}})=0.35. \end{eqnarray*}

Based on these results, we summarize similar and same communities in Table 2. Similar communities are those whose similarity scores are higher than 0. Note that although c3 is similar to c1 and c2, c3 does not represent a closest community to any of the older communities c1 and c2, since c4 and c5 score higher similarity scores when compared to c2 and c3. Also note that our CC function is bi-directional, so we can also say that, for example, c1 is closest to c4.

**Table 2:** An example of similar and same communities.

Community	Similar to	Closest to
c3	c1 and c2	/
c4	c1	c1
c5	c2	c2

## Results and Analysis

In the following sections we present statistics for identified (closest) communities and their main hub ontologies, present results of validation with MEDLINE abstracts, analyse transition (evolution) between different time points, analyse the coherence of communities, and present results of measuring effects of ontology sizes on community detection.

### Statistics, identified communities and their hub ontologies

Table 3 shows statistics for all five versions of our graphs. The values in the first column are as follows:

**Table 3:** Statistics for different versions of the graph.

	Oct12	Feb13	Aug13	Dec13	Jul14
MAV	0.346	0.339	0.343	0.435	0.402
#All	283	294	317	359	367
#Map	254	268	259	321	318
%Map	90%	91%	82%	89%	87%
#NoMap	29	26	58	38	49
%NoMap	10%	9%	18%	11%	13%
#Com	5	6	7	7	6
#C1	87	127	88	211	160
#C2	85	54	46	49	65
#C3	31	35	43	28	48
#C4	31	20	39	11	30
#C5	20	28	36	11	12
#C6	/	4	5	7	3
#C7	/	/	2	4	/

**Notes.**

Abbreviations are as follows: MAV modularity analysis value #All number of all ontologies #Map/#NoMap number of ontologies with at least one/no mappings %Map/%NoMap percentage of ontologies with at least one/no mappings #Com number of communities #Cx community x

• MAV represents Modularity Analysis values,
• #All is the number of all ontologies in the graph,
• #Map is the number of ontologies with at least one mapping (source or target),
• %Map is percentage of ontologies with at least one mapping (source or target),
• #NoMap is the number of ontologies with no mappings,
• %NoMap is the percentage of ontologies with no mappings,
• #Com is the number of identified communities. The #Cx (0 < *x* < 8) values represent the number of ontologies in each identified community. The number of ontologies in each community orders communities.

**Figure 2:**
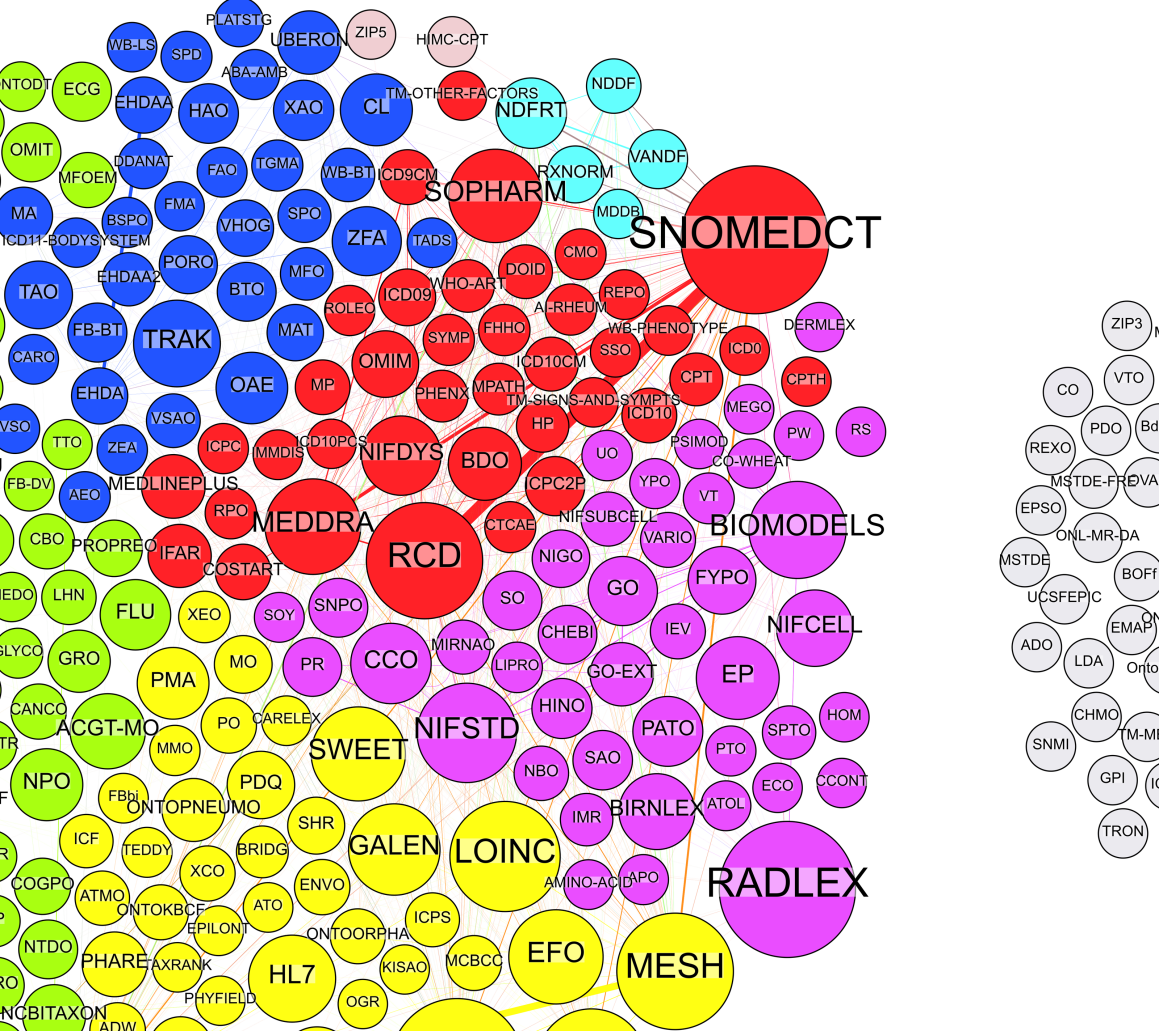
Illustration of identified communities in a graph. Different colours represent communities, while nodes represent ontologies. Node labels are ontology abbreviations and node sizes correspond to BC values. Grey nodes are ontologies with no mappings.

The MA values in Table 3 are all below 0.5 with highest being the last two versions. Low MA values indicate that it is difficult to identify well-structured and independent communities between BioPortal ontologies. We can notice that the number of all ontologies rises over time, which is a result of new ontologies being added to the repository. The proportion of mapped ontologies indicates that the majority of new ontologies have no mappings. The number of identified communities changed over time from five identified communities in Oct12 to six or seven identified communities in later versions. Figure 2 illustrates a part of identified communities from the August 2013 data, where each colour represents different community, and each node represents an ontology. Node size correspond to ontology BC values. We present changes in these communities (e.g., ontologies switching their communities, i.e., changing the colour in the graph) in the Transition analysis section.

Table 4 shows ontologies with the highest BC values (i.e., main hub ontologies) for each community (communities are again ranked by their size). SNOMEDCT (Systematized Nomenclature of Medicine—Clinical Terms) is the ontology with the highest overall BC score (ignoring the communities) in each version, which makes it the most important hub ontology. There are several reasons for that. First, SNOMEDCT contains other ontologies (e.g., RCD) and extensive sub terminologies that we expect to find represented in other ontologies. Next, according to BioPortal’s webpage, SNOMEDCT is also the first most viewed ontology with 50% more views than NDF (National Drug File), which is on the second place. Finally, SNOMEDCT has also been identified the most prominent hub ontology with Ghazvinian’s methods (Ghazvinian et al., 2009).

In the next section we align the communities and discuss their changes between consecutive graphs.

### Validating the communities with MEDLINE

**Figure 3:**
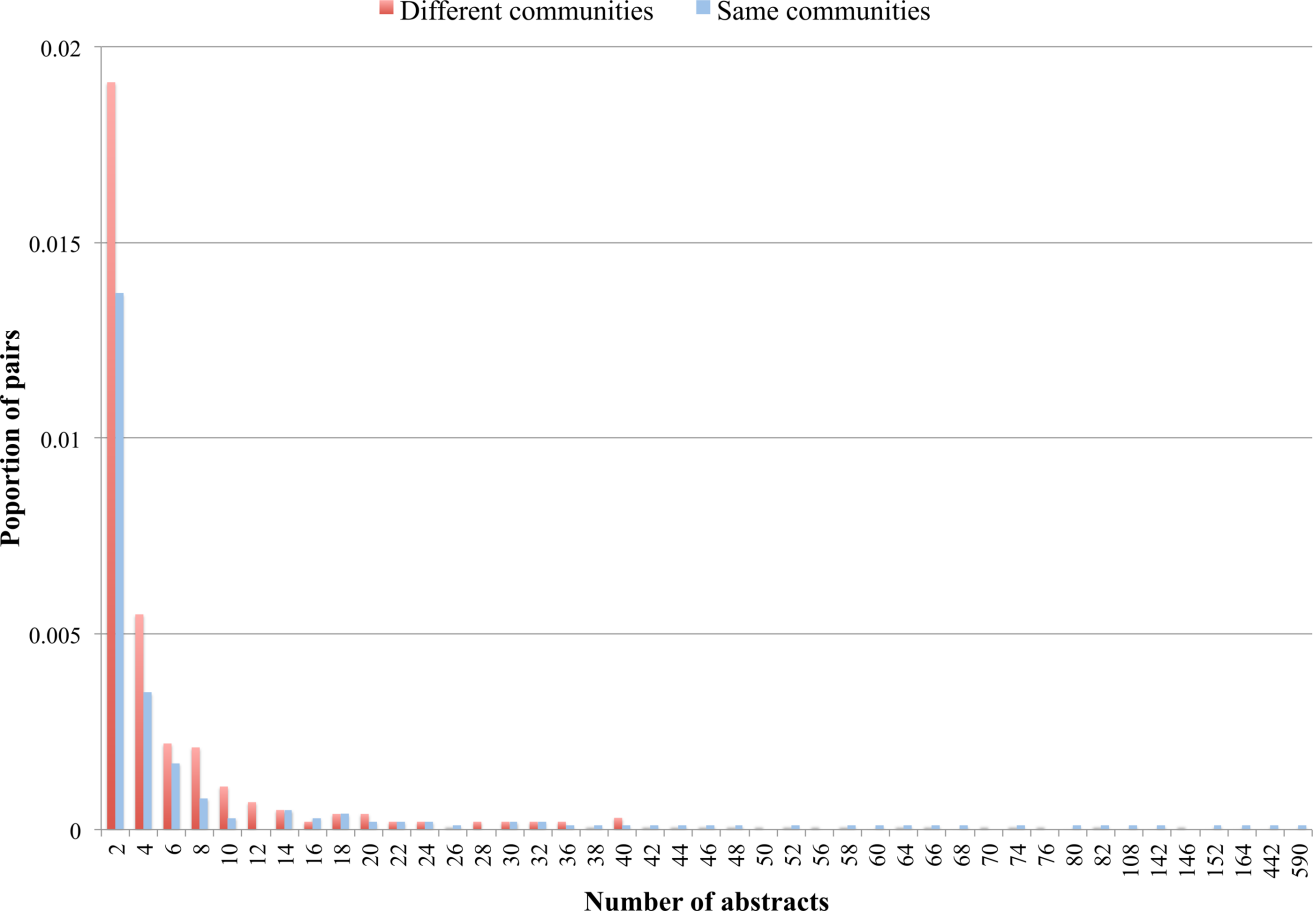
Proportion of ontology pairs found in different number of abstracts The horizontal axis displays the number of abstracts, while the vertical axis displays the proportion of ontology pairs for each number of abstracts.

We found 3,020 ontology pairs (less than 3% of all possible pairs) that were mentioned together in at least one abstract. Figure 3 shows proportions of ontology pairs found in at least 2 MEDLINE abstracts where each ontology is from either a different (red) or the same (blue) community. Although the differences on Fig. 3 are small (*Y* axis), we can notice that pairs where each ontology belongs to a different community tend to be found in lower number of abstracts (i.e., from 2 to 10 abstracts). On the other hand, ontology pairs that can be found together in large numbers of abstracts (e.g., 108, 152 or 164 abstracts) tend to belong to the same community.

**Table 4:** Identified communities and their main hub ontologies for all versions of the graph. Please note that the communities are not aligned.

	Oct12	Feb13	Aug13	Dec13	Jul14
**1**	EP	NIF	ERO	NCIT	SWEET
**2**	NCIT	NCIT	NCIT	**SNOMEDCT**	**SNOMEDCT**
**3**	UBERON	UBERON	RADLEX	NIFSTD	NIFSTD
**4**	RADLEX	RADLEX	**SNOMEDCT**	BIOMODELS	SYN
**5**	**SNOMEDCT**	**SNOMEDCT**	TRAK	MESH	MESH
**6**	/	NCBITaxon	NDFRT	NCBITaxon	SWO
**7**	/	/	HIMC-CPT	SWO	/

**Notes.**

Abbreviations EP Cardiac Electrophysiology Ontology NCIT National Cancer Institute Thesaurus UBERON Uber Anatomy Ontology RADLEX Radiology Lexicon SNOMEDCT Systematized Nomenclature of Medicine—Clinical Terms NIF Neuroscience Information Framework NCBITaxon National Center for Biotechnology Information Organismal Classification ERO Eagle-I Research Resource Ontology TRAK Taxonomy for Rehabilitation of Knee Conditions NDFRT National Drug File—Reference Terminology SWO Software Ontology SYN Sage Bionetworks Synapse Ontology SWEET Semantic Web for Earth and Environment Technology Ontology

**Figure 4:**
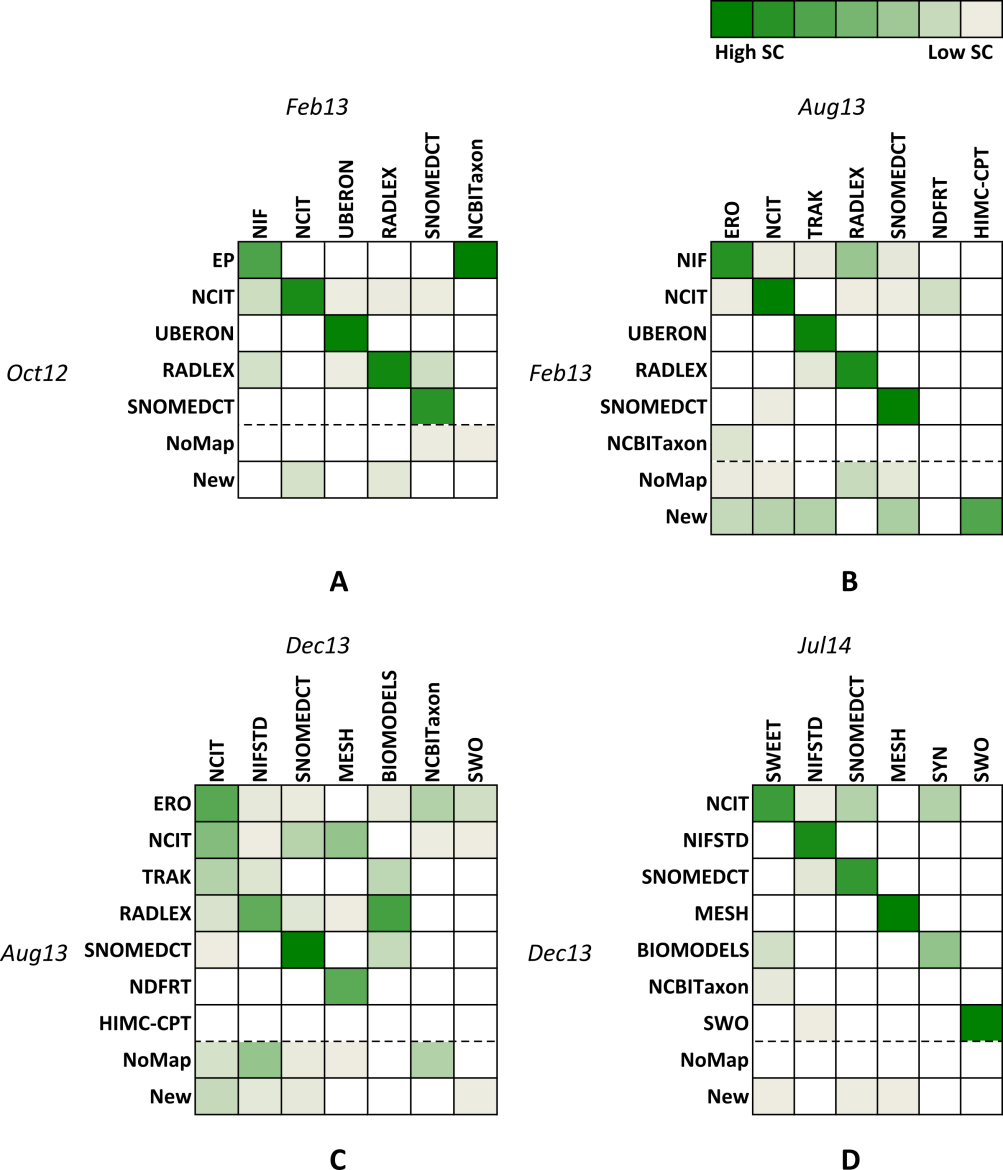
Similarities between graph pairs. (A) Feb13 vs Oct12, (B) Aug12 vs Feb13, (C) Dec13 vs Aug 13, and (D) Jul14 vs Dec13. Column and row names represent the main hub ontology for each identified community. Different shades of green correspond to similarity scores where darker colours represent higher numbers and lighter colours represent lower numbers. The NoMap row represents ontologies that had no mappings in the previous version of the graph but are members of one of the communities in the newer version. The New row represents ontologies that did not exist in the previous version of the graph.

These results imply that identified communities contain ontologies that appear more often together in the literature. However, since most ontologies pairs were not found in the abstracts, different methods should be explored (e.g., citations to ontologies, analysing full texts, similarity matching). This is an area for future investigations.

### Transition analysis

Figure 4 represents four heat maps for similarities between pairs of consecutive graph versions. Column and row names represent the main hub ontology for each identified community. Columns contain names for recent versions, while rows contain names for older versions. Different shades of green correspond to similarity scores where darker colours represent higher numbers and lighter colours represent lower numbers. White colour corresponds to the similarity score of zero and shows communities with no similarity. The *NoMap* row represents ontologies that had no mappings in the previous version of the graph but are members of one of the communities in the newer version. The *New* row represents ontologies that did not exist in the previous version of the graph.

When observing heat maps on Fig. 4, we can see how communities evolve over time. Observing single columns indicates how many older communities or their parts merge into a single newer community (e.g., Red and Blue communities in Fig. 2 could merge into one community in the future). On the other hand, observing single rows indicates into how many new communities an older community splits (e.g., Red community in Fig. 2 could become two communities in the future).

For example, let us consider the heat map A (we can interpret B, C and D in the similar way). Row names represent hub ontologies for the old version (Oct12), while column names represent ontologies for the new version (Feb 13) of the graph. The third row shows that all ontologies from the UBERON community stayed in the same community, i.e., the community did not split. On the other hand, observing row 2 shows that although the majority of Oct12 NCIT ontologies stayed in the closest community in Feb13 (i.e., NCIT, column 2), some ontologies also migrated into the NIF (column 1), UBERON (column 3), RADLEX (column 4) and SNOMEDCT (column 5) communities. The third column illustrates merging of parts of three different communities (i.e., NCIT, UBERON and RADLEX) into the new UBERON community. The heat map A also shows that the new identified community (i.e., NCBITaxon, last column) mainly consists of ontologies from the old EP (row 1) community and some ontologies that had no mappings in Oct12 (row 6).

With the heat maps we can find pairs of closest communities, which are identified with the most intensive shades of green in each column. For example, on the heat map A, the NIF column contains three coloured squares. However, the square in the EP row is the most intensive shade of green, which identifies the closest community to NIF. In Table 5 we align identified closest communities and their corresponding main hub ontologies in groups from G1 to G6.

**Table 5:** Aligned closest communities, and their main hub ontologies.

	Oct12	Feb13	Aug13	Dec13	Jul14
G1	EP	NIF	ERO	NCIT	SWEET
G2	SNOMEDCT	SNOMEDCT	SNOMEDCT	SNOMEDCT	SNOMEDCT
G3	RADLEX	RADLEX	RADLEX	NIFSTD	NIFSTD
G4	NCIT	NCIT	NCIT	MESH	MESH
G5	UBERON	UBERON	TRAK	BioModels	SYN
G6	/	/	/	SWO	SWO

**Figure 5:**
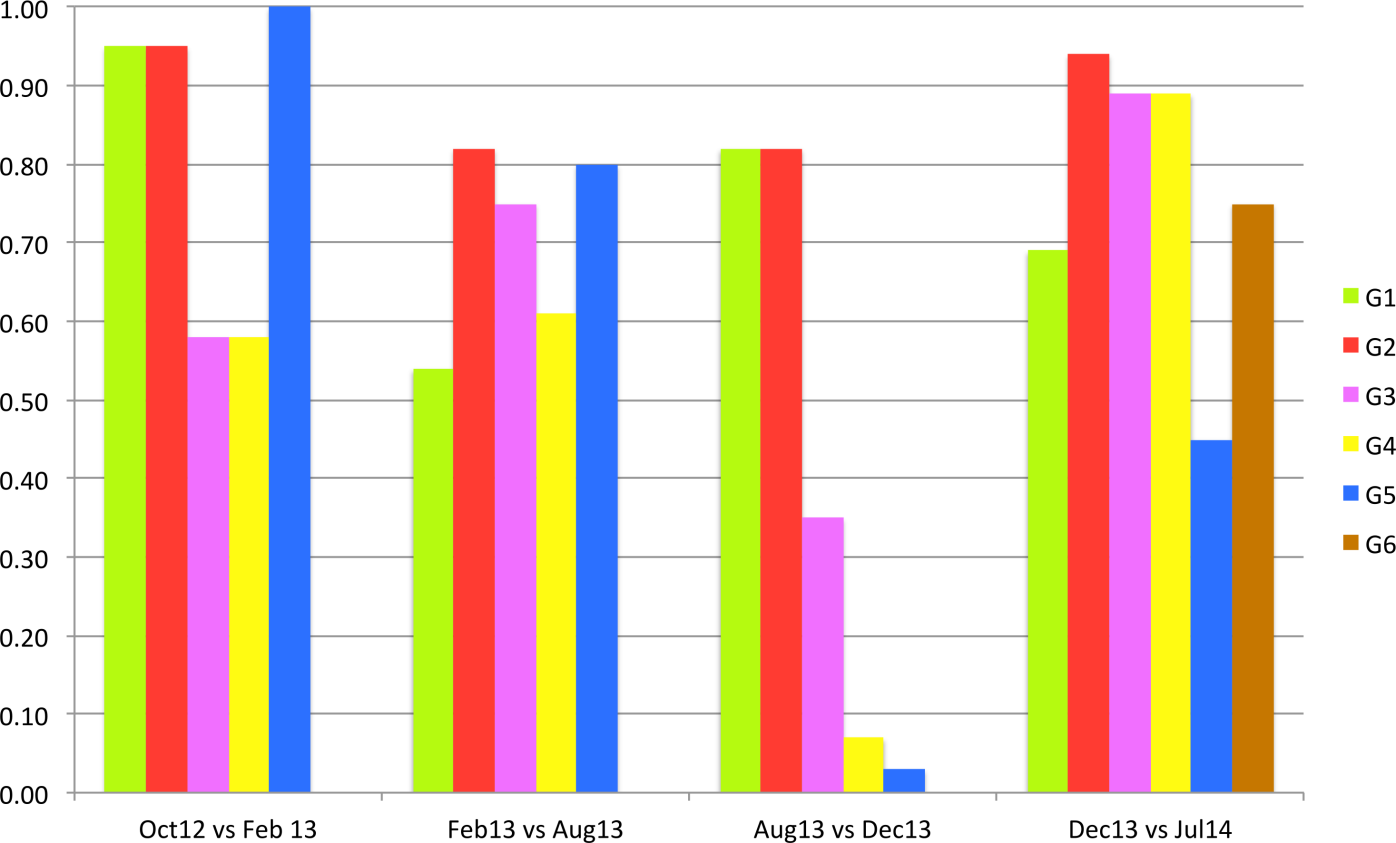
Proportion of ontologies that stay in the same closest community between graph pairs.

Figure 5 shows the proportion of ontologies that “stay” in each group of the closest communities between two consecutive versions of the graph. As we notice, we identified six groups of the closest communities, where five of them keep more than half of ontologies in the first three versions of the graph. In Dec13, G1 and G2 keep majority of ontologies, while G3, G4 and G5 lose more than half of the ontologies. In the latest version only G5 loses more than half of the ontologies, while other communities keep the majority of their ontologies.

Table 5 and Fig. 5 show that some closest communities keep the same core ontologies over several versions of graph (e.g., G2 and G3), while other closest communities contain different core ontologies for each version of the graph (G1 and G5). We could say that G2 group represents the most stable group over all versions of the graph. Figure 4 shows that more than 90% of the G2 ontologies stayed in the closest community in Feb13 and Jul14, and 80% of G2 ontologies stayed in the same community in Aug13 and Dec13. SNOMEDCT is G2’s core ontology for all versions of the graph.

Figure 5 also shows that three groups of closest communities lost more than half of their ontologies in Dec13 with two G4 and G5 loosing more than 90% of their ontologies. When comparing these results with heat map C on Fig. 1, we notice that the majority of these ontologies joined the largest community (the first column and the second and third rows). Closer analysis of mapping data showed that many new mappings have been added to BioPortal in Dec13, which uses an updated version of BioPortal data, i.e., BioPortal 4. The latter was a major update of the portal that used largely updated data. Some of the mapping information is significantly different when comparing to older versions. For example, RADLEX had been core ontology in all the versions before December 2013. However, in the latest version this ontology has only a few mappings. Also, it is interesting that the latest two versions result in highest MAVs (Table 3), which indicates that ontologies might be clustered better compared to previous versions. It will be interesting to see if this affects stability in the future.

An interesting community is the NCBITaxon community, which appears the Feb13 and Dec13 versions. We already learned that some taxonomy ontologies formed their own community in February 2013 (Kocbek et al., 2013). However, this community merged with the largest community in Aug13 (Fig. 1). The community was identified again in Dec13, but then again merged in Jul14.

Considering Fig. 4 and the heat map D one can notice that communities in the last two versions keep most ontologies compared to previous versions. This indicates that the mapping data changed the least compared to previous data and BioPortal gained in stability.

### Analysing community coherence

**Figure 6:**
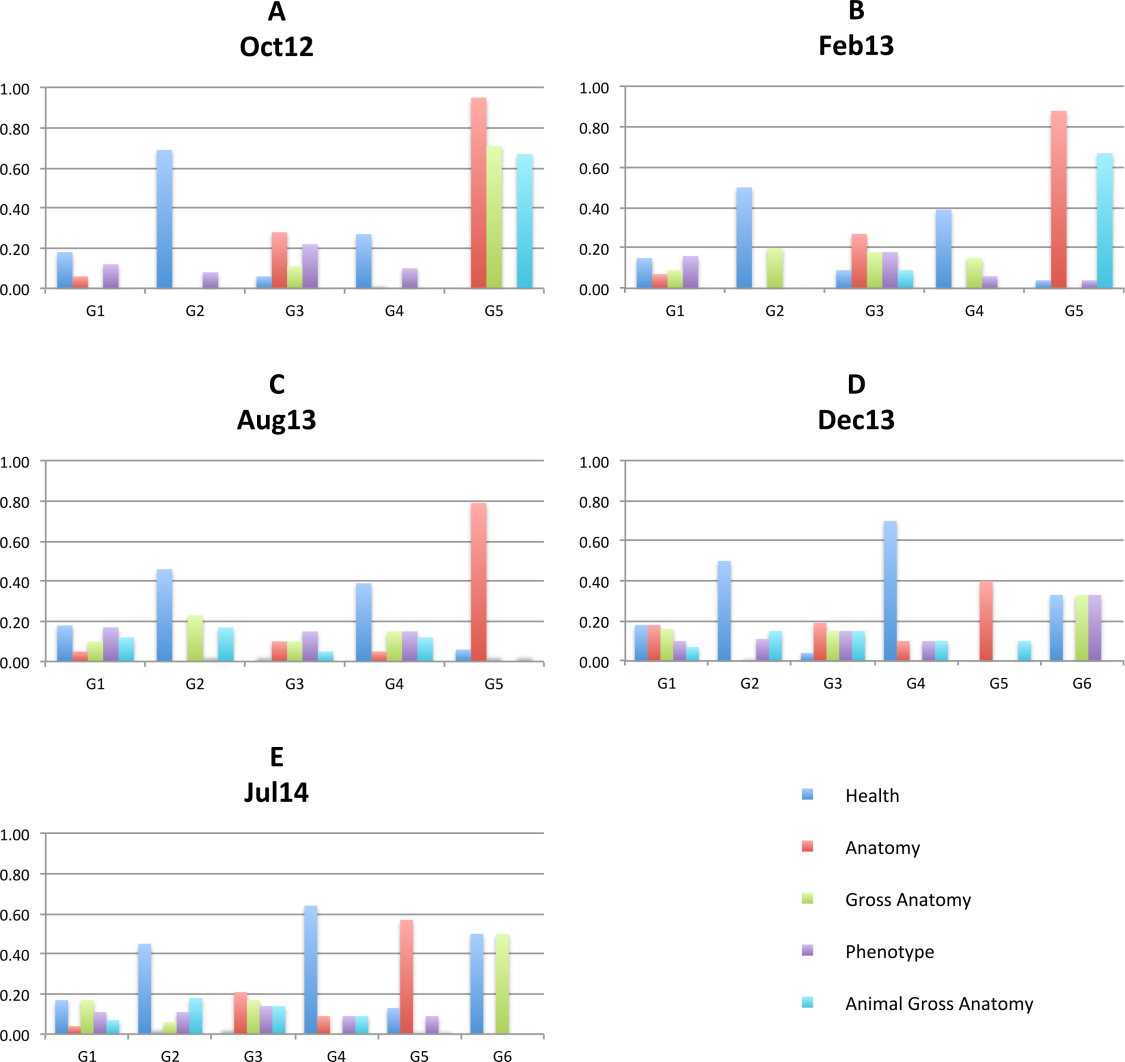
Top 5 categories. Distribution of top 5 categories with highest number of members (Health, Anatomy, Gross Anatomy, Phenotype, Animal Gross Anatomy) for all 5 graph versions: Oct 12 (A), Feb13 (B), Aug13 (C), Dec13 (D), Jul14 (E).

BioPortal groups ontologies into 41 *categories* such as Anatomy, Health, Ethology, and Gene Product. In addition, information about *projects* that use BioPortal ontologies is available. We use these two types of information to discuss the coherence of identified communities in our methods. Figure 6 illustrates distribution of top 5 categories with highest number of members (Health, Anatomy, Gross Anatomy, Phenotype, Animal Gross Anatomy) for all 5 graph versions. The horizontal axis present closest communities and the vertical axis present ratio of community members belonging to each category.

Charts on Fig. 6 show that all identified communities contain ontologies from different categories for all graph versions. However, we can notice that more than 90% of G5 ontologies in the Oct12 version belong to the Anatomy category and around 70% of G5 ontologies belong to the Gross Anatomy and Animal Gross Anatomy categories. In the future versions, G5 still contains the largest proportion of anatomy ontologies. Again, the Dec13 version shows major changes with large drop of anatomy ontologies in G5. We analysed mapping data for two ontologies that were in G5 in Aug13, i.e., Foundational Model of Anatomy (FMA) and Mosquito Gross Anatomy Ontology (TGMA). The former stayed in G5 also in Dec 13, while the latter switched to G1. We observed large increase of overlapping ontologies for both ontologies in Dec13. A large number of newly overlapping ontologies for TGMA belongs to other communities, which is probably the reason for its migration.

Another distinct community is G2, where a large proportion of ontologies belongs to the Health category. Almost 70% of Oct12 G2 ontologies are categorised as health ontologies and present the majority in G2 future graphs as well. Health ontologies are distributed through other identified communities in the future and present large portions of G4 and G6. Communities G1 and G3 are more heterogeneous with a mixture of ontologies from all categories in all graph versions.

We also investigated the BioPortal project data to analyse its alignment with identified communities. Each project has a list of ontologies that it uses and we investigated how these lists correspond to the identified communities for the Jul14 version. Project with the highest number of ontologies used 35 ontologies, while the majority of projects used a single ontology. We ignored the latter projects in our analysis since it was obvious that they will be aligned with a single community. Table 6 shows number or projects using ontologies from only 1, 2, 3, 4, 5 or 6 identified communities for 77 projects that use at least two ontologies. We can notice that most projects use ontologies from 2 or 3 identified communities. However, 12 projects use ontologies from the same community. In addition, some projects use the majority of ontologies from the same community. For example, G5, which contains most of anatomy ontologies as we discussed above, provides all ontologies for a database containing genomic and biological information on anopheline mosquitoes (i.e., the AnoBase project (Topalis et al., 2005)). G5 also contains 8 out of 10 ontologies for the Bgee database (Bastian et al., 2008), which compares expression patterns between animals. Bgee creates homology relationships between anatomical ontologies, and stores this information in a multi-species ontology. These examples show that our clusters contain all or the majority of ontologies being used in narrower projects.

**Table 6:** Number of connected ontologies in each graph version for two anatomy ontologies.

#Communities	1	2	3	4	5	6
#Projects	12	26	27	7	5	0

### Analysing the effect of ontology sizes on community detection

An important factor that influences the number of mappings between two ontologies is the size (i.e., number of classes) of both ontologies. It is more likely that larger ontologies have higher number of mappings when compared to smaller ontologies. Unfortunately, we did not collect ontology sizes for each time spot in our analysis and historical data is not available through BioPortal’s API. We downloaded old versions of ontologies at the time of writing this paper and tried to manually parse the ontologies with the OWL API to calculate their sizes. The OWL API is a Java API and reference implementation for creating, manipulating and serialising ontologies (Horridge & Bechhofer, 2011). However, due to issues such as missing ontology imports, parsing errors, and license restrictions, we were unable to calculate correct sizes for a large number of ontologies. Ignoring these ontologies would not produce comparable results with our previous analysis. To address this problem, we gathered mapping information and ontology sizes for November 2015 and produced two new graphs. In the first graph, we applied the same community detection techniques as described in the previous sections, while in the second graph, we normalised number of mappings by ontology sizes.

Table 7 shows results for two graphs using data gathered in November 2015 with two different sources for edge weights: (a) number of mappings, and (b) number of mappings normalised by ontology sizes. Both graphs result in 6 identified communities with the same hub ontologies. Between the two graphs, two communities are completely identical, while other three communities result in minor changes. Specifically, out of 437 ontologies, 12 ontologies (i.e., approx. 3%) change the communities. None of these ontologies were hub ontologies. These findings imply that ontology sizes do not play an important role in community detection for our data. However, we plan to investigate these findings in more depth in future graph versions.

**Table 7:** Comparison of community information for November 2015 with and without considering ontology size.

Size	MAV	#Ontologies	#Comm	#C1	#C2	#C3	#C4	#C5	#C6
No	0.346	437	6	255	107	30	26	12	7
Yes	0.339	437	6	259	106	29	24	12	7

## Discussion and Conclusion

In this paper we focused on investigating a comprehensive repository of biomedical ontologies (BioPortal) using graph theory concepts. We performed the exploratory study of BioPortal’s mapping data over different time points. As far as we know, this is the first attempt of this kind. With investigating mapping data gathered at five different time points using graph theory methods, we identified similar and closest communities of overlapping ontologies, and demonstrated evolution of communities over time. We also tried to validate communities through mentions of their ontology members in MEDLINE abstracts.

The five communities identified in the first version of the graph changed their size. We showed how communities appear, disappear, split or merge over time. Based on similarity scores we determined closest communities between pairs of different graph versions. We then analysed the stability of these closest communities. We discussed how identified communities align with BioPortal’s category and project information. We also identified core ontologies of the closest communities.

When studying our conclusions, we should take into consideration some limitations of the work. First, the BioPortal repository can be publicly modified and no evaluation of the uploaded ontologies or mapping data is done. In addition, although we tried to identify them, there are probably some “test” ontologies left in our data. Therefore, we should expect some data noise. Our analysis also showed large differences in data between the Aug13 and Dec13 when BioPortal 4 was announced. Second, our method for identifying communities might favour larger ontologies since we do not consider ontology sizes when calculating edge weights. Although our analysis of data gathered in November 2015 implies that normalising edge numbers results in small changes in final graph, this remains an area for future investigation. Next, due to limitations of BioPortal’s web service API, we were not able to distinguish between different types of ontology mappings in older versions. For example, the MESH and RH-MESH ontologies have same concepts and only differ in syntactic translation, which has not been picked up by our methods. Finally, our observations highly depend on the Louvain method for community detection. We accept this method as a “ground truth” quality metrics of our clusters. The Louvain method was the only available method in Gephi and it is considered as the fastest and most accurate method in terms of modularity score (Aynaud & Guillaume, 2010).

In the future, we plan to address the above issues, especially distinguishing between different types of mappings and considering ontology sizes. We also plan to consider other graph centrality measures and methods for community detections. Finally, we plan to perform a deeper analysis of changes in the underlying ontologies to investigate how these affect the broader graph clustering patterns.

##  Supplemental Information

10.7717/peerj.2990/supp-1 Supplemental Information 1 Ontology mapping information Mappings between pairs of ontologies for different versions of graphs.

## Additional Information and Declarations

### Data Deposition

The following information was supplied regarding data availability: The raw data has been supplied as Supplemental Information 1.
